# Investigating the Mechanism of Action of *Ipomoea pes-caprae* in the Treatment of Rheumatoid Arthritis Based on Serum Metabolomics and Network Pharmacology

**DOI:** 10.3390/md23030114

**Published:** 2025-03-07

**Authors:** Fangfei Zhong, Siwei Li, Xianglong Pan, Juan Wen, Jinling Xie, Zhengcai Du, Erwei Hao, Jiagang Deng, Xiaotao Hou

**Affiliations:** 1 Guangxi Key Laboratory of Efficacy Study on Chinese Materia Medica, Guangxi University of Chinese Medicine, Nanning 530200, China; zhongff63@126.com (F.Z.); 13597086313@163.com (S.L.); panxl2885@163.com (X.P.); wenj20132@gxtcmu.edu.cn (J.W.); 13257716536@163.com (J.X.); duzhengcai8@163.com (Z.D.); ewhao@163.com (E.H.); 2 University Engineering Research Center of Reutilization of Traditional Chinese Medicine Resources, Guangxi University of Chinese Medicine, Nanning 530200, China; 3 Guangxi Key Laboratory of TCM Formulas Theory and Transformation for Damp Diseases, Guangxi University of Chinese Medicine, Nanning 530200, China; 4 Department of Pharmaceutical, Heilongjiang University of Chinese Medicine, Harbin 150040, China

**Keywords:** rheumatoid arthritis, *Ipomoea pes-caprae*, serum metabolomics, network pharmacology, pharmacokinetics, mechanism of action

## Abstract

*Ipomoea pes-caprae* (L.) Sweet (Convolvulaceae) is a commonly used marine Chinese medicine in the coastal areas of southern China. Traditionally, it has been used in the treatment of rheumatoid arthritis (RA). However, the mechanism of action against RA remains unclear. This study aimed to explore the mechanism of action of *Ipomoea pes-caprae* water extract (IPE) in the treatment of RA through serum metabolomics and network pharmacology. Rat models of RA with wind-dampness cold bi-syndrome (WCM) and wind-dampness heat bi-syndrome (WHM) were established to evaluate the therapeutic effect of IPE against RA. Ultra-high performance liquid chromatography-quadrupole time-of-flight mass spectrometry (UPLC-Q-TOF-MS/MS) technology was used to analyze the absorbed components of IPE in the plasma of the two models. Serum metabolomics was employed to identify potential biomarkers and metabolic pathways of IPE in the treatment of RA. The key targets and related pathways of RA were screened using network pharmacology and validated using molecular docking. The biomarker-pathway-target network was mapped via the combination of metabolomics and network pharmacology. A total of 10 chemical constituents were identified from WHM rat plasma, and eight chemical constituents were identified from WCM rat plasma. Serum metabolomics research identified 20 endogenous potential biomarkers, and 10 major metabolic pathways closely related to WHM and WCM. Network pharmacology analysis yielded 65 overlapping targets, with the core targets being ALB, AKT1, EGFR, and CASP3. Molecular docking showed that the four absorbed components in plasma had a strong binding activity with ALB and AKT1. Combining metabolomics and network pharmacology, two major biomarkers and two major pathways were identified. IPE can effectively relieve the symptoms of RA, and the potential mechanism of IPE in treating RA has been preliminarily elucidated. These results can provide a scientific basis for further drug research and development, as well as clinical application.

## 1. Introduction

Rheumatoid arthritis (RA) is a systemic inflammatory autoimmune disease characterized by synovial hyperplasia, synovial inflammation, and joint destruction. It belongs to the category of ‘bi-syndrome’ in traditional Chinese medicine (TCM) [1]. Bi-syndrome is a syndrome of pain, soreness, numbness, weight, and movement disorder of limbs and joints caused by feeling the pathogenic factors of wind, cold, dampness, and heat. As the disease progresses, the incidence of limb disability and limited physiological function increases [2], so early diagnosis and intervention are crucial. At present, there is a lack of specific treatment measures for RA in clinical practice, and drug treatment is still the main treatment. The treatment goals are to reduce pain, control the development of inflammation, delay disease progression, and maintain joint function to the greatest extent. The most common types of drugs include non-steroidal anti-inflammatory drugs (NSAIDs), corticosteroids (CORT), disease-modifying anti-rheumatic drugs (DMARDs), etc. Although these drugs can effectively relieve the symptoms and progression of the disease, they still cannot fundamentally treat the disease and may cause toxic side effects such as cardiovascular diseases, damage of gastrointestinal tract, as well as liver and kidney [3]. Therefore, there is an urgent need to discover safer and more effective drugs for clinical treatment of RA. TCM has attracted much attention from researchers as a key component of complementary and alternative therapies due to its powerful therapeutic effects and relatively few side effects. Modern studies have shown that TMC has significant efficacy and low side effects in the treatment of various diseases, highlighting its importance in the modern medical system [4,5,6]. TCM treatment of bi-syndrome focuses on syndrome differentiation and treatment. According to the patient’s individual differences, symptoms, constitution, etiology, and other factors, personalized treatment plans are formulated for syndrome differentiation and treatment [7]. This individualized treatment can improve the patient’s symptoms and quality of life more comprehensively. In addition, TCM emphasizes the holistic concept and pays attention to regulating body functions as a whole. In the treatment of RA, it not only focuses on the inflammation and pain of local joints, but it also considers the overall condition of the whole body, such as immune function and endocrine status. Through multi-target and multi-way, the homeostasis of various physiological systems in the body is restored as a whole. TCM is increasingly being valued [8].

Serum metabolomics is an approach to exploring biological systems from a holistic perspective. It reflects the physiological and metabolic status of organisms by analyzing changes in metabolites in the body [9,10]. This is of great significance for the early diagnosis of diseases, the understanding of pathological mechanisms, and the discovery of new drugs. Network pharmacology is an integrative pharmacology that uses systems biology, bioinformatics, and network science methods to construct a visualized network of “drug-target-disease” to reveal the complex mechanism of TCM acting on diseases [11]. It is helpful to understand the comprehensive effects of multi-component and multi-target drugs and promote the development of personalized medicine and precision medicine [12]. In short, the combination of these two approaches in TCM research can more comprehensively and systematically reveal the overall pharmacodynamic characteristics and pharmacological mechanisms of TCM.

*Ipomoea pes-caprae* (L.) Sweet, belonging to the Convolvulaceae family and the *Ipomoea* genus, is a herbaceous climbing plant widely distributed in tropical and subtropical regions. It is one of the most common beach plants in the world. In the southern coastal areas of China, *Ipomoea pes-caprae* is a commonly used traditional marine medicine among the Jing ethnic group. In addition, *Ipomoea pes-caprae*, as a marine TCM, is also widely popular in tropical and subtropical countries such as Thailand, Brazil, Mexico, and Australia. It has the effects of dispelling wind and eliminating dampness, resolving abscesses, and dispersing nodules, as well as drawing out toxins and reducing swelling. It is commonly used to treat lumbar muscle strain and rheumatic joint pain [13,14,15]. Several marine-derived compounds can be effectively used to treat rheumatoid arthritis [16]. Marine TCM is rich in medicinal resources. The chemical constituents of *Ipomoea pes-caprae* mainly include resin glycosides, terpenoids, flavonoids, phenolic acids, volatile compounds, steroids, and other compounds, which exhibit various biological activities such as anti-inflammatory, antiviral, antitumor, antibacterial, and immunomodulatory effects [17]. The anti-inflammatory activity of *Ipomoea pes-caprae*, which aligns with its traditional efficacies, is considered one of its primary pharmacological effects. Our previous study demonstrated that *Ipomoea pes-caprae* has significant anti-RA efficacy [18] and good therapeutic effects on rats in the models of wind-dampness cold bi-syndrome (WCM) and wind-dampness heat bi-syndrome (WHM) [19]. However, its mechanism of action in the treatment of RA has not been fully elucidated. In the present study, we carried out the first metabolomics analysis of *Ipomoea pes-caprae* water extract (IPE) in a rat model of RA disease to obtain the differences in metabolic responses between the two models of bi-syndrome, followed by the combination of metabolomics and network pharmacology to identify the key anti-RA proteins in IPE. Finally, we successfully revealed the pharmacological basis, metabolic pathway, potential target, and mechanism of action of the IPE in the treatment of RA, which provides a scientific basis for its further drug development and clinical application.

## 2. Results

### 2.1. Effects of IPE on AI Score and Left Hind Toe Swelling in RA Rats

**Figure 1:**
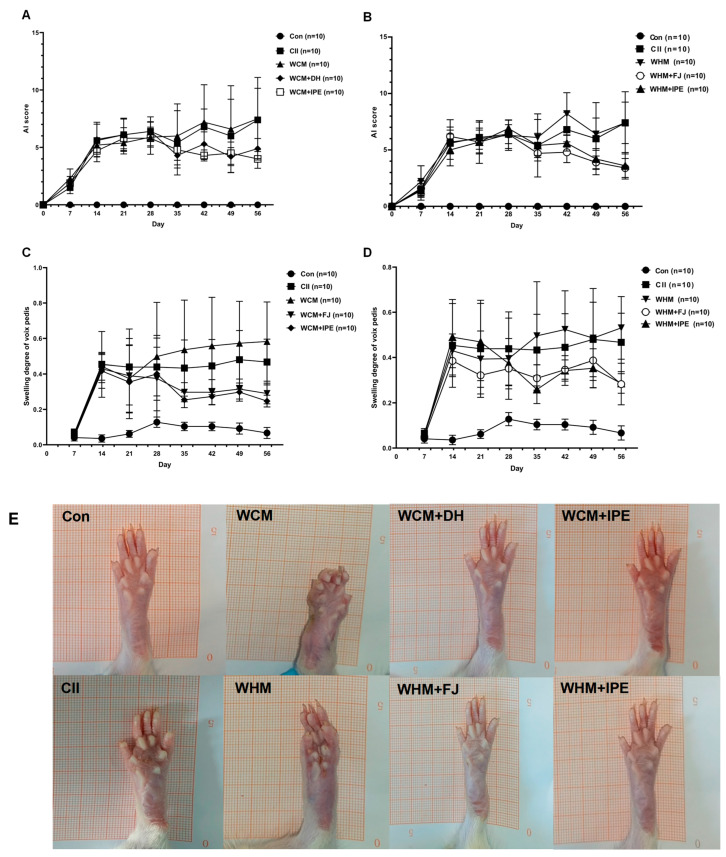
Effect of IPE on AI score and left hind toe swelling in rats. (**A**): Trend plot of AI scores in WCM rats; (**B**): trend plot of AI scores in WHM rats; (**C**): trend plot of left hind toes swelling in WCM rats; (**D**): trend plot of left hind toes swelling in WHM rats; (**E**): representative macroscopic photographs of left hind toes swelling of each groups. Normal control group (Con); natural bovine type II collagen model group (CII); wind-dampness cold bi-syndrome model group (WCM); wind-dampness heat bi-syndrome model group (WHM); wind-dampness cold bi-syndrome + Duhuo group (WCM + DH): Wind-dampness heat bi-syndrome + Fangji group (WHM + FJ); wind-dampness cold bi-syndrome + *Ipomoea pes-caprae* group (WCM + IPE); wind-dampness heat bi-syndrome + *Ipomoea pes-caprae* group (WHM + IPE).

IPE showed significant therapeutic effects on both WCM and WHM rats. As shown in Figure 1A,B, compared with the normal control group (Con), the Arthritis Index (AI) scores of rats in each model group increased statistically significantly after seven days of modeling, which was statistically significant (*p* < 0.01). Compared with the WHM group, when administered on the 28th day, the AI scores of rats in the wind-dampness heat bi-syndrome + Fangji group (WHM + FJ) and wind-dampness heat bi-syndrome + *Ipomoea pes-caprae* group (WHM + IPE) decreased (*p* < 0.05 or *p* < 0.01). When compared with the WCM group, the AI scores of rats in the wind-dampness cold bi-syndrome + Duhuo group (WCM + DH) and the wind-dampness cold bi-syndrome + *Ipomoea pes-caprae* group (WCM + IPE) decreased (*p* < 0.05 or *p* < 0.01) on the 28th day of administration. The comparison of AI score of rats in each group is shown in Table 1.

In addition, as shown in Figure 1C,D, compared with the Con group, rats in each model group exhibited significant toe swelling on day 14 after modeling, and the difference was statistically significant (*p* < 0.01). On the 35th day of the experiment, it could be observed that the rats in the WCM and WHM groups showed more severe swelling of the left hind toe compared with the CII, but there was no significant difference. Compared with the WHM group, on the 14th day of drug administration, the swelling degree of the left hind toe of rats in the WHM + FJ group and WHM + IPE group decreased statistically significantly (*p* < 0.05 or *p* < 0.01). Compared with the WCM group, the swelling of the left hind toe of rats in the WCM + DH group and WCM + IPE group decreased statistically significantly (*p* < 0.05 or *p* < 0.01) on the 14th day of drug administration. The comparison of left hind toes swelling of rats in each group is shown in Table 2.

### 2.2. Pathological Section of Ankle Synovial Tissue and Micro-CT of the Ankle Joint of Rats

**Figure 2:**
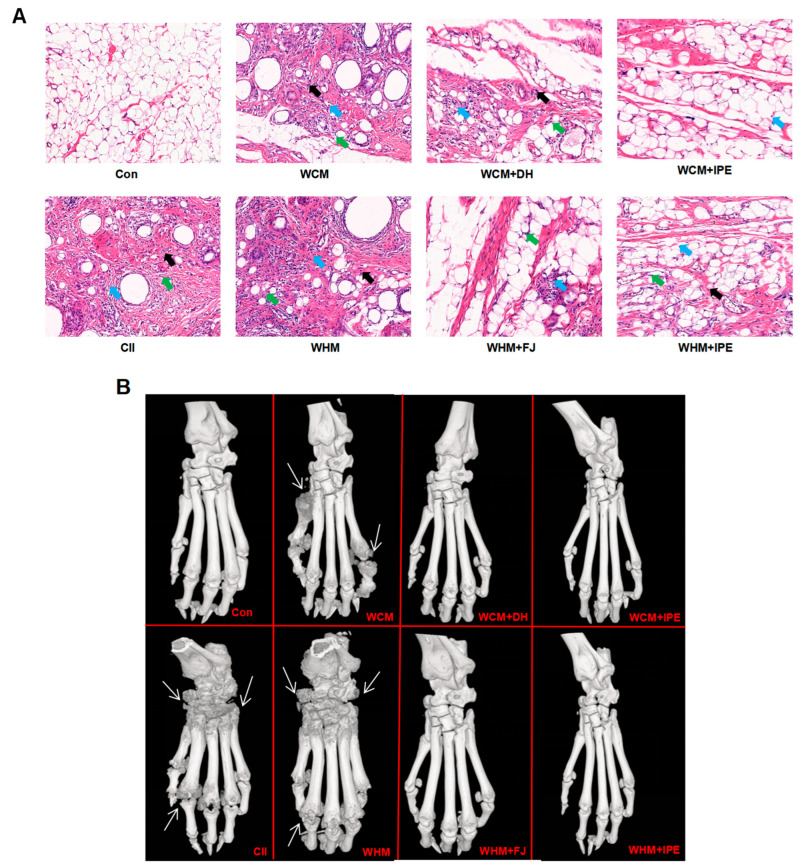
Effects of IPE on the pathological changes in ankle joints of rats (**A**), micro-CT images (**B**). Blue arrows: lymphocytes; black arrows: fibroblasts; green arrows: fiber cells; white arrows: bone destruction; normal control group (Con); natural bovine type II collagen model group (CII); wind-dampness cold bi-syndrome model group (WCM); wind-dampness heat bi-syndrome model group (WHM); wind-dampness cold bi-syndrome + Duhuo group (WCM + DH); wind-dampness heat bi-syndrome + Fangji group (WHM + FJ); wind-dampness cold bi-syndrome + *Ipomoea pes-caprae* group (WCM + IPE); wind-dampness heat bi-syndrome + *Ipomoea pes-caprae* group (WHM + IPE).

According to the pathological section of ankle synovial tissue of rats, compared with the Con group, the degree of synovial tissue lesions in rats in each model group was relatively severe, and inflammatory cell infiltration and fibrous tissue proliferation were observed in the synovial tissue, with significant differences. Compared with the WHM group, the degree of synovial tissue lesions in the WHM + FJ group and WHM + IPE group was relatively reduced. Compared with the WCM group, the degree of synovial tissue lesions was also relatively reduced in the WCM + DH group and WCM + IPE group (Figure 2A). In addition, according to micro-computed tomography (micro-CT), it could be observed that the ankle joints of the model rats in each group showed bone damage, irregular articular bone structure, joint space narrowing, severe erosion, and other changes in the joints compared with the Con group. After administration of IPE intervention, the ankle joint damage in rats in all groups showed different degrees of improvement (Figure 2B).

### 2.3. Effect of IPE on Inflammatory Factors in Serum

**Figure 3:**
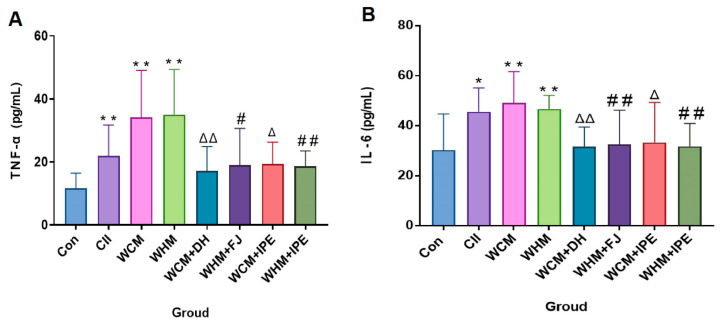
Effect of IPE on the expression levels of inflammatory factors TNF-α (**A**) and IL-6 (**B**) in serum. Normal control group (Con); natural bovine type II collagen model group (CII); wind-dampness cold bi-syndrome model group (WCM); wind-dampness heat bi-syndrome model group (WHM); wind-dampness cold bi-syndrome + Duhuo group (WCM + DH); wind-dampness heat bi-syndrome + Fangji group (WHM + FJ); wind-dampness cold bi-syndrome + *Ipomoea pes-caprae* group (WCM + IPE); wind-dampness heat bi-syndrome + *Ipomoea pes-caprae* group (WHM + IPE). The data were analyzed by one-way analysis of variance (ANOVA) followed by the Student–Newman–Keuls post hoc test. For each category (*n* = 10), * *p* < 0.05, ** *p* < 0.01, significantly different from the Con; ^△^ *p* < 0.05, ^△△^ *p* < 0.01, significantly different from the WCM; ^#^ *p* < 0.05, ^##^ *p* < 0.01, significantly different from the WHM.

Compared with the Con group, the TNF-α and IL-6 levels of rats in the CII and the WCM and WHM groups increased significantly with statistical significance (*p* < 0.01), and the TNF-α and IL-6 levels of rats in the WCM and WHM groups were higher than those in the CII. Compared with the WHM group, the TNF-α and IL-6 levels of rats in the WHM + FJ group and WHM + IPE group decreased significantly (*p* < 0.05 or *p* < 0.01). Compared with the WCM group, the TNF-α and IL-6 levels decreased in rats in the WCM + DH group and WCM + IPE group (*p* < 0.05 or *p* < 0.01), as shown in Figure 3A,B.

### 2.4. Characterization of the Chemical Constituents of IPE

**Figure 4:**
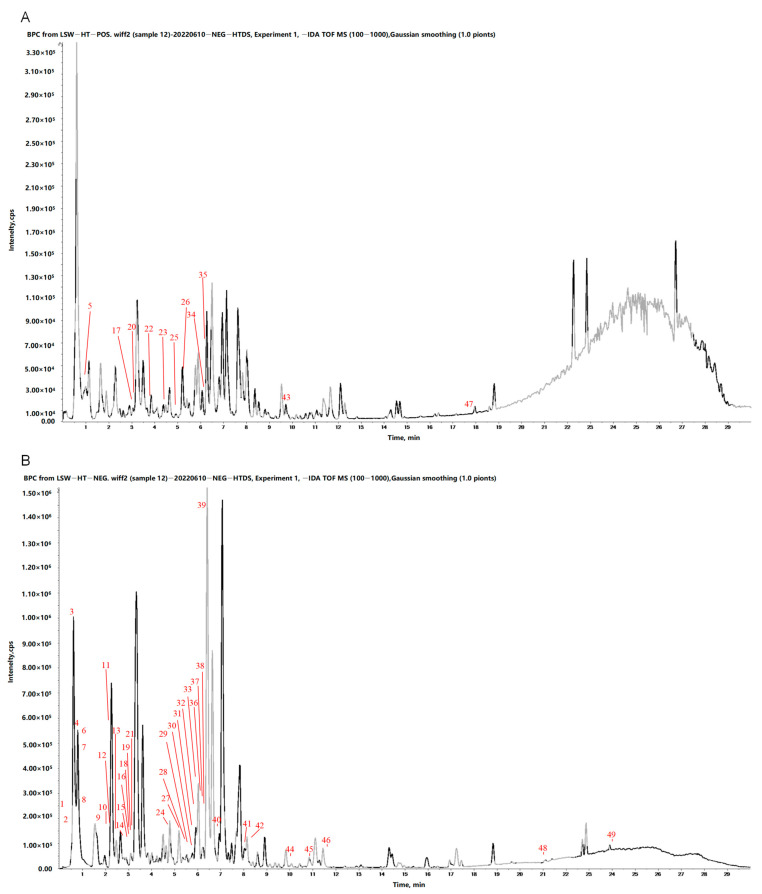
Base peak chromatogram of IPE. (**A**): positive ions; (**B**): negative ions. Peak No 1~49 are compounds corresponding to Table 3 “Paek No.” 1~49, respectively.

In this study, 49 chemical components were identified from IPE by ultra-performance liquid chromatography-mass spectrometry (UPLC-MS), including one resin glycoside, nine flavonoids, 28 organic acids, five coumarins, one lignan, two nucleosides, two terpenes, and one vitamin. The positive and negative base peak chromatogram (BPC) of IPE are shown in Figure 4A,B. The molecular formulae, secondary fragment ions, and relative content information for each of the 49 identified chemical components are presented in Table 3.

### 2.5. Identification and Relative Contents of the Absorbed Components in Plasma of the Con + IPE, WCM + IPE, and WHM + IPE Groups

Firstly, plasma samples from the Con, Con + IPE, WCM, WHM, WCM + IPE, and WHM + IPE groups were analyzed by UPLC-MS in positive and negative ion modes, respectively. Second, the precise relative molecular mass and secondary fragmentation information of the absorbed components in plasma were compared with the IPE component identification results to further identify the absorbed prototype of the components in plasma. The results showed that eight absorbed components in plasma were initially identified from the Con + IPE group, including caffeic acid, p-hydroxybenzoic acid, ferulic acid, isoscopoletin, scopoletin, methyl caffeate acid, protocatechuic acid, and esculetine. From the WHM + IPE group, 10 absorbed components in plasma were initially identified, including caffeic acid, p-hydroxybenzoic acid, ferulic acid, isoscopoletin, scopoletin, methyl caffeate acid, coumarin, protocatechuic aldehyde, protocatechuic acid, and esculetine. Eight absorbed components in plasma were initially identified from the WHM + IPE group, where these eight absorbed components in plasma were consistent with those of the Con + IPE group (Table 4).

### 2.6. Pharmacokinetic Analysis of the Absorbed Components of IPE in the Plasma

**Figure 5:**
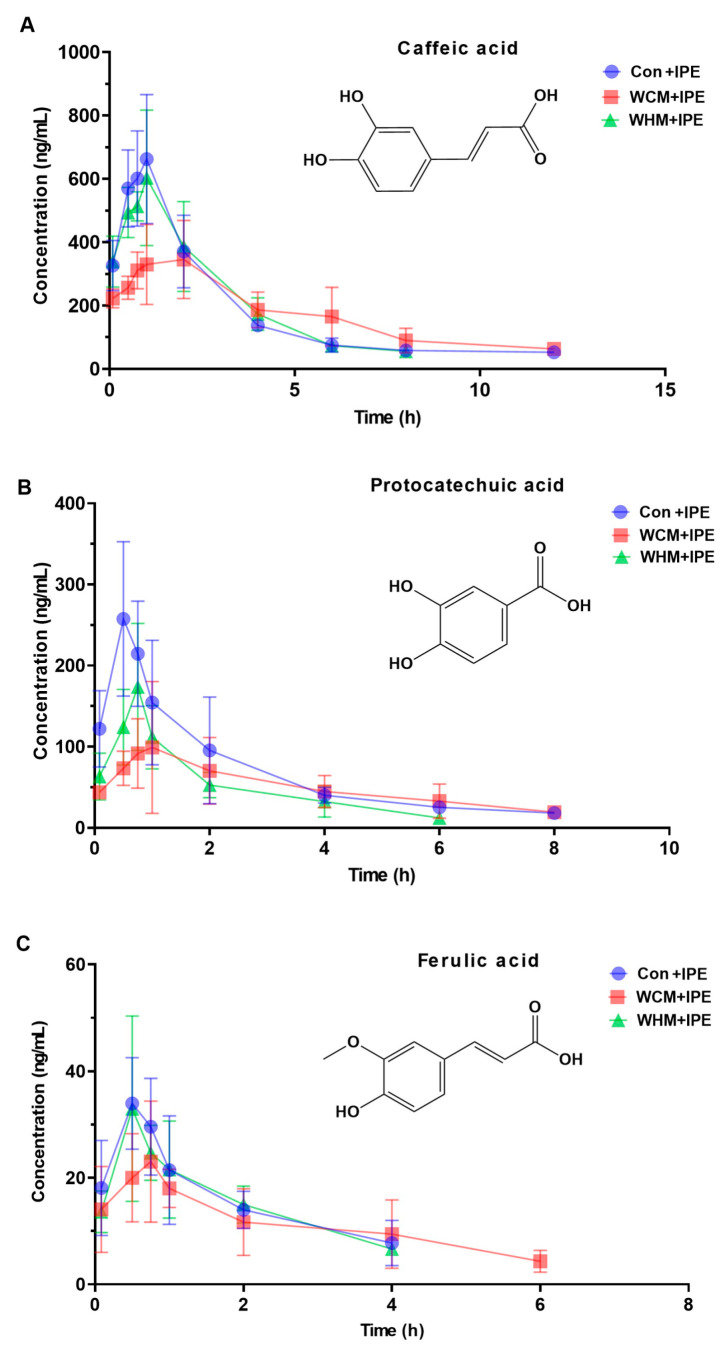
Pharmacokinetic profiles of caffeic acid (**A**), protocatechuic acid (**B**), and ferulic acid (**C**) components of IPE in rats ($\bar{x}$ ± s, *n* = 6). Normal control + *Ipomoea pes-caprae* group (Con + IPE); Wind-dampness cold bi-syndrome + *Ipomoea pes-caprae* group (WCM + IPE); Wind-dampness heat bi-syndrome + *Ipomoea pes-caprae* group (WHM + IPE).

In the preliminary stage of this study, we carried out a comprehensive analysis of the differential content of IPE in different models and administration groups and finally chose caffeic acid, protocatechuic acid, and ferulic acid among the absorbed components of IPE in the plasma to further investigate the in vivo pharmacokinetic characteristics of IPE. The results showed that the area under the drug–time curve (AUC_0~t_) of the three absorbed components in the plasma was smaller in the WCM + IPE and WHM + IPE groups compared with that in the Con + IPE group; the time to peak drug concentration (T_max_) and half-life (t_1/2_) of the absorbed components in the plasma were prolonged in the WCM + IPE group; the t_1/2_ of the blood concentration was shortened in the WHM + IPE group; and the T_max_ was comparable in the WHM + IPE and Con + IPE groups; compared with WHM + IPE, the WCM + IPE group had lower peak concentration (C_max_) and longer t_1/2_, indicating that there were differences in the rate and extent of absorption of different components of IPE in normal and pathological model rats. The drug–time curves of caffeic acid are shown in Figure 5A, protocatechuic acid in Figure 5B, and ferulic acid in Figure 5C. The main pharmacokinetic parameters are shown in Table 5.

### 2.7. Serum Metabolomics Analysis of IPE in RA Rats

#### 2.7.1. PCA of Serum Samples

**Figure 6:**
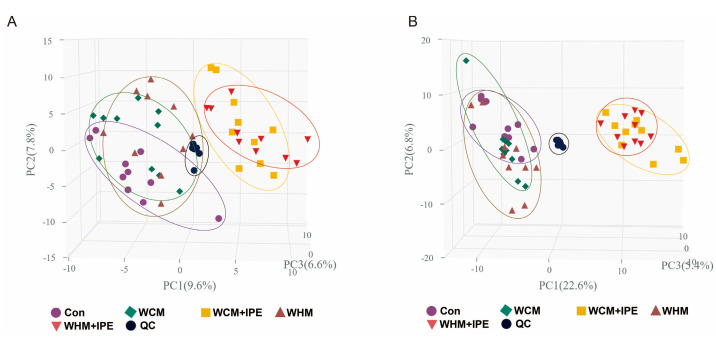
Plot of PCA scores of serum samples in rats. (**A**): positive ion mode, (**B**): negative ion mode. Normal control group (Con); wind-dampness cold bi−syndrome model group (WCM); wind−dampness heat bi−syndrome model group (WHM); wind−dampness cold bi−syndrome + *Ipomoea pes-caprae* group (WCM + IPE); wind-dampness heat bi−syndrome + *Ipomoea pes−caprae* group (WHM + IPE); quality control (QC) samples.

The results of multivariate data analysis are shown in Figure 6A,B. First, unsupervised principal component analysis (PCA) analysis was performed on each experimental group, including the Con, WCM, WHM, WCM + IPE, and WHM + IPE groups. The quality control (QC) samples clustered well in positive and negative ion modes, indicating that the instrumental method was applicable. The normal control group and each model group were well distinguished in both modes, indicating that the RA model was successfully constructed. The administered group differed from each model group in both modes, indicating that IPE has a certain therapeutic effect on RA.

#### 2.7.2. OPLS-DA Analysis of Serum Samples

**Figure 7:**
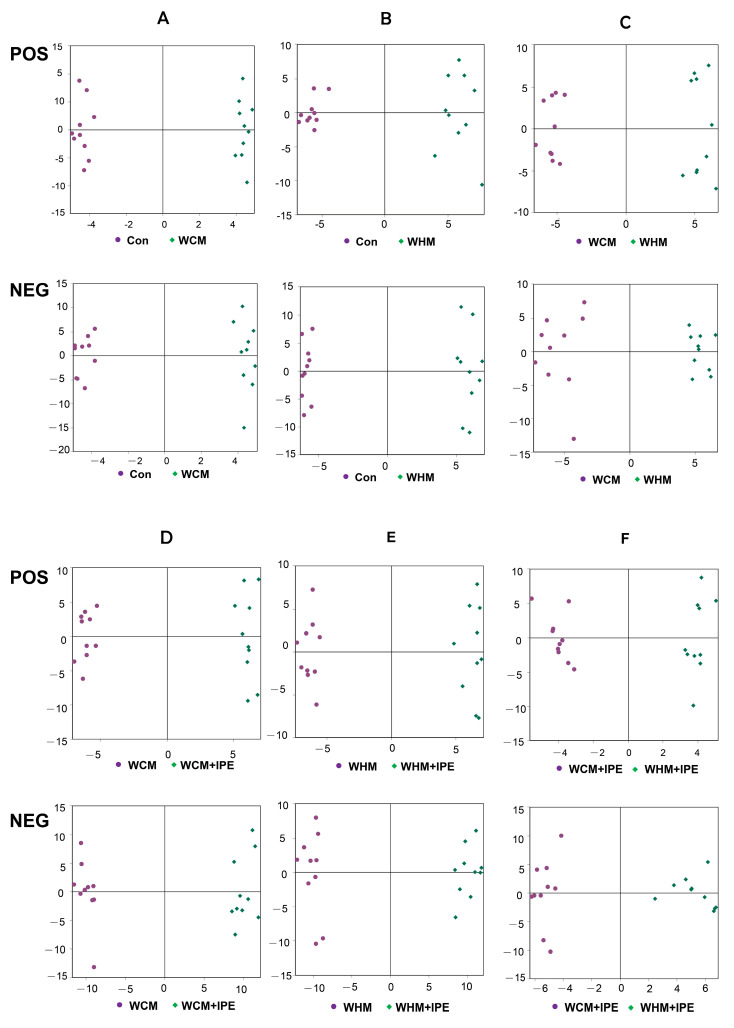
OPLS-DA model scores of serum positive and negative ions in rats. (**A**): Normal control group (Con) vs. wind−dampness cold bi−syndrome model group (WCM); (**B**): normal control group (Con) vs. wind−dampness heat bi−syndrome model group (WHM); (**C**): wind−dampness cold bi−syndrome model group (WCM) vs. wind-dampness heat bi−syndrome model group (WHM); (**D**): wind-dampness cold bi-syndrome model group (WCM) vs. wind-dampness cold bi-syndrome + *Ipomoea pes−caprae* group (WCM + IPE); (**E**): wind−dampness heat bi−syndrome model group (WHM) vs. Wind−dampness heat bi−syndrome + *Ipomoea pes−caprae* group (WHM + IPE); (**F**): wind−dampness cold bi−syndrome + *Ipomoea pes−caprae* group (WCM + IPE) vs. wind−dampness heat bi−syndrome + *Ipomoea pes−caprae* group (WHM + IPE).

The data of the Con, WCM, and WHM groups were analyzed by OPLS-DA modeling in positive and negative ion modes, respectively, and the score plots are shown in Figure 7. As can be seen from the figure, the model was not overfitted in the positive and negative ion modes, with good explanatory power and reliable data analysis. In the positive and negative ion modes, the Con, WCM, and WHM groups were separated in terms of composition, and the endogenous composition of rat serum was changed after modeling, which indicated that the modeling was successful. In the comparison between the WCM and WHM groups, the composites were separated, indicating that there might be differences between the two modeling groups. Compared with the WCM and WHM groups, the WCM + IPE and WHM + IPE groups were separated by components, indicating that IPE administration interfered with the metabolism of serum endogenous components. The inter-group comparison between the WCM + IPE and WHM + IPE groups showed separation of components, suggesting that IPE may exert its efficacy by regulating different endogenous metabolites.

#### 2.7.3. Identification of Endogenous Biomarkers

A total of 11 potential biomarkers closely related to wind-dampness heat bi-syndrome and 10 potential biomarkers closely related to wind-dampness cold bi-syndrome were screened and identified under positive and negative-ion modes. The results showed that the serum levels of 1-methyl-l-histidine were significantly higher (*p* < 0.05), while the levels of urocanic acid, D-allose, D-threonate, hippuric acid, L-threonate, pentachlorophenol, pyrocatechol, creatinine, DL-normetanephrine, and malonic acid were significantly lower (*p* < 0.05, *p* < 0.01) in rats from the WHM group compared with those from the Con group. Compared with the WHM group, the content of each component in the serum of rats in the WHM + IPE group was significantly adjusted back (*p* < 0.05, *p* < 0.01). Compared with the Con group, the serum levels of capric acid, O-succinyl-L-homoserine, octanoic acid, uracil, γ-aminobutyric acid, 2′-deoxycytidine, and cytosine in rats of the WCM group were significantly increased (*p* < 0.05), while the levels of urocanic acid, p-coumaryl alcohol, and D-mannose were significantly reduced (*p* < 0.05, *p* < 0.01). Compared with the WCM group, the content of each component in the serum of rats in the WCM + IPE group was significantly restored (*p* < 0.05, *p* < 0.01). The results indicated that IPE had a good modulatory effect on the endogenous biomarkers in the serum of rats with WCM and WHM (Table 6).

#### 2.7.4. Analysis of the Major Metabolic Pathways in IPE

**Figure 8:**
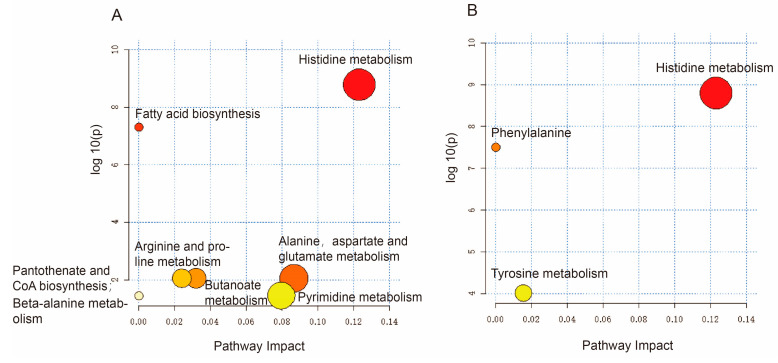
Enrichment analysis of metabolic pathways of IPE in rats with anti-RA wind-dampness cold bi-syndrome and wind-dampness heat bi-syndrome. (**A**): Disturbance metabolic pathway of wind-dampness cold bi-syndrome; (**B**): disturbance metabolic pathway of wind-dampness heat bi-syndrome.

IPE administration was able to significantly affect eight metabolic pathways in wind-dampness cold bi-syndrome: histidine metabolism, fatty acid biosynthesis, alanine, aspartate and glutamate metabolism, butanoate metabolism, arginine and proline metabolism, pyrimidine metabolism, and pantothenate and CoA biosynthesis, beta-alanine metabolism. IPE administration could significantly affect three metabolic pathways in wind-dampness heat bi-syndrome, namely histidine metabolism, phenylalanine metabolism, and tyrosine metabolism. As shown in Figure 8A,B.

### 2.8. Network Pharmacological Analysis of IPE Against RA

**Figure 9:**
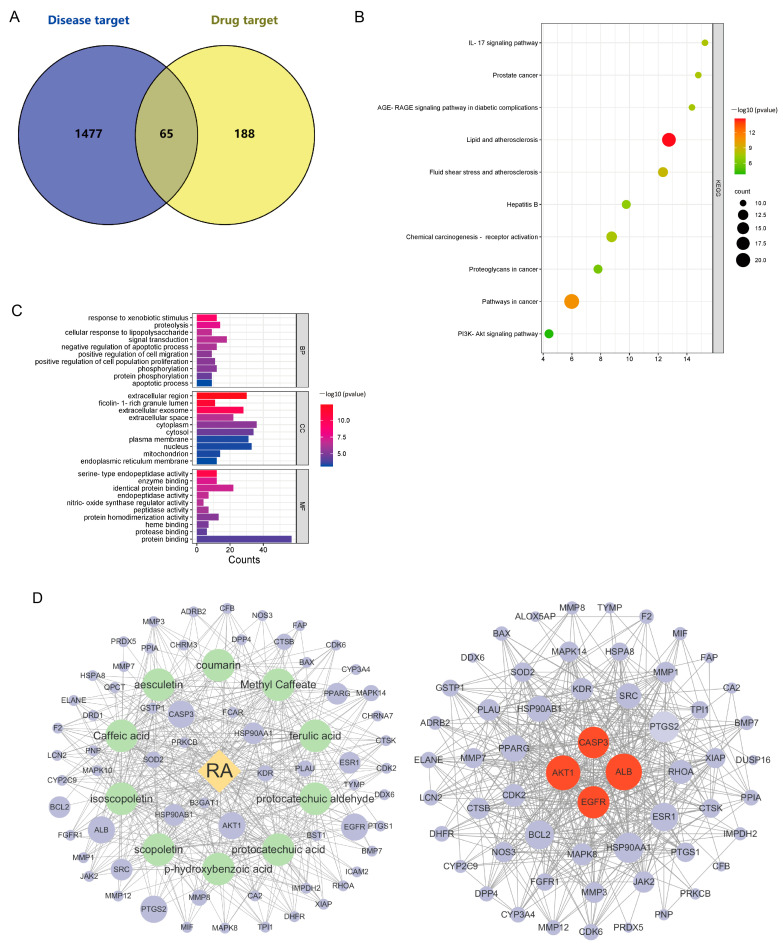
Network pharmacology analysis of IPE anti-RA. (**A**): Venny diagram of disease-drug intersection targets; (**B**): KEGG-enriched top 10 pathway bubble diagram; (**C**): GO functional enrichment analysis gradient level bar graph; (**D**): “compound-target-disease” interaction network diagram and protein–protein interaction network. The red circle represents the core target, the green circle represents the absorbing component, the purple circle represents the relevant potential target, and the orange square represents the disease. Larger circles indicate higher degree values.

In this experiment, 10 components absorbed components of IPE in the plasma by the UPLC-MS technique were identified as the potential bioactive components of IPE, which might form the possible pharmacological material basis for IPE to exert its pharmacological effects against RA with wind-dampness cold bi-syndrome and wind-dampness heat bi-syndrome. Based on the 10 absorbed components of IPE in the plasma, 253 drug targets were predicted from the PharmMapper database and TCMSP database. Combined with Gene Cards and OMIM databases, 1542 RA disease targets were obtained. From the Venn diagram of “disease target-drug target” overlap, 65 targets were identified as potential targets for IPE in RA (Figure 9A). KEGG pathway enrichment analysis showed that the IL-17 and PI3K/Akt signaling pathways were the most critical signaling pathways among the top 10 signaling pathways (Figure 9B). GO analysis was performed to enrich the biological processes (BPs), cellular components (CCs), and molecular functions (MFs) of the protein targets, as shown in Figure 9C. An “absorbed components-target-disease” interaction network was constructed, which consisted of 75 nodes and 432 edges. The core targets were calculated by CentiScaPe 2.2, and the center nodes with a high degree of association were ALB, AKT1, EGFR, and CASP3, suggesting that these were the key targets (Figure 9D). Table 7 lists the core targets and their degree values.

### 2.9. Molecular Docking of Absorbed Components in the Plasma to Key Targets

**Figure 10:**
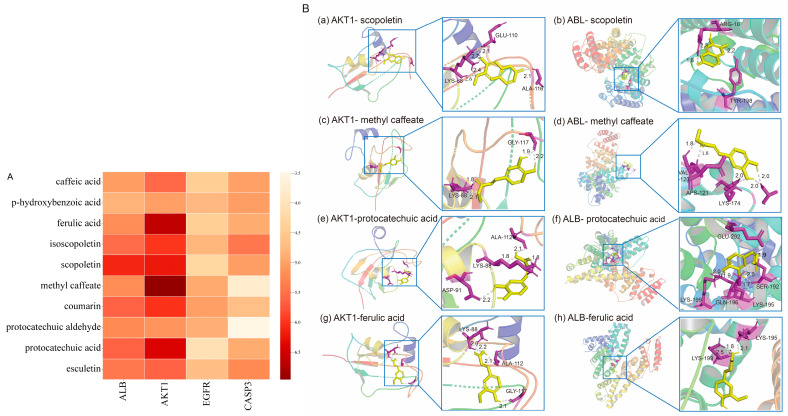
Molecular docking of absorbed components of IPE in the plasma to key targets. (**A**): Heat map of molecular docking; (**B**): visualization of molecular docking of components to targets.

A total of 10 absorbed components in the plasma were subjected to molecular docking with the four key targets sequentially, and the results are shown in Figure 10A. The depth of color represents the absolute value of binding energy. The darker the color, the greater the absolute value of binding energy and the stronger the binding effect. The combinations with stronger binding effects were visualized (Figure 10B). The results showed that the binding energy of scopoletin docked with AKT1 was −6.23 kJ/mol, forming three hydrogen bonds with residue ALA116 of AKT1; the binding energy of scopoletin docked with ALB was −6.11 kJ/mol, forming two hydrogen bonds with residue ARG186 of ALB and one hydrogen bond with residue TYR138. Methyl caffeate docked with AKT1 had a binding energy of −6.96 kJ/mol, and formed two hydrogen bonds with residue GLY117 of AKT1; methyl caffeate docked with ALB had a binding energy of −5.20 kJ/mol, and formed four hydrogen bonds with residues ASP121, VAL120, LYS174, and ALA172 of ALB. The binding energy of protocatechuic acid docked with AKT1 was −6.4 kJ/mol, and five hydrogen bonds were formed with ASP91 residue of AKT1; the binding energy of protocatechuic acid docked with ALB was −5.66 kJ/mol, and three hydrogen bonds were formed with LYS-199, GLN-196, and SER-192 residues of ALB; and the binding energy of ferulic acid docked with AKT1 was −6.63 kJ/mol, forming four hydrogen bonds with the LYS-88, ALA-112, and GLY-117 residues of AKT1; the binding energy of ferulic acid docked with ALB was −5.18 kJ/mol, forming three hydrogen bonds with the LYS-199 and LYS-195 residues of ALB.

### 2.10. Network Analysis of Endogenous Biomarkers and Targets

**Figure 11:**
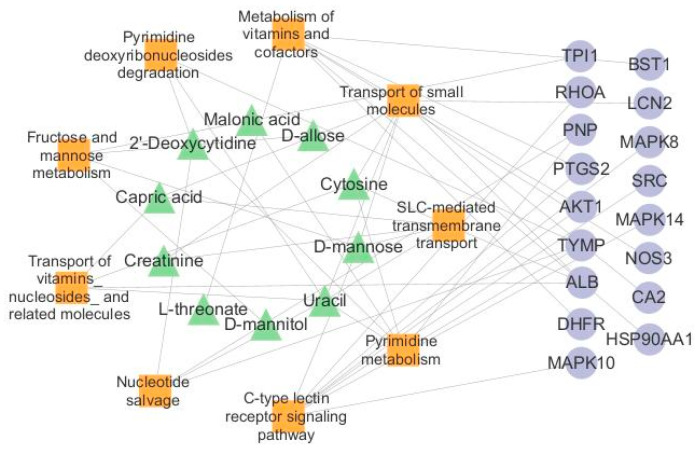
Biomarker-pathway-target network diagram of IPE anti-RA. Green triangles represent biomarkers, orange squares represent pathways, and purple circles represent targets.

A total of 20 endogenous biomarkers identified by metabolomics and 65 potential targets obtained by network pharmacology were jointly analyzed, and the results were visualized using Cytoscape 3.10 to construct a visual network diagram of biomarkers-pathways-targets. The results of the analysis showed the interactions of nine pathways, 17 targets, and 10 biomarkers, with a total of 36 nodes (Figure 11). The final further screening yielded two key signaling pathways: SLC-mediated transmembrane transport pathway and C-type lectin receptor signaling pathway; key endogenous biomarkers as D-mannose and cytosine; core targets as ALB and AKT1.

## 3. Discussion

The pathogenesis of RA is quite complex, and one of the core links is the persistent chronic inflammation of joint synovium induced by pro-inflammatory cytokines. In this process, inflammatory factors such as IL-6 and TNF-α play a crucial role and are regarded as key factors in the inflammatory process of RA. Studies have shown that the levels of pro-inflammatory cytokines, such as IL-6 and TNF-α, significantly up-regulate in patients with RA [20]. In particular, IL-6 not only serves as one of the major mediators of systemic inflammation in RA but also plays a key pro-inflammatory role in the pathogenesis of the disease and regulates joint pain [21]. Notably, the present experimental data showed that IPE could effectively reduce the serum levels of IL-6 and TNF-α, thereby inhibiting the progression of RA to a certain extent.

X. Cheng et al. explored the mechanism of *Ipomoea pes-caprae* in AIA arthritis rats under network pharmacological analysis [22]. Compared with the AIA model, the CIA model is a gold standard model for exploring the pathogenesis of RA and evaluating therapeutic drugs, which has been widely recognized and applied [23]. In this study, in order to follow the theory of TCM, the principle of identification and treatment, and to further validate the traditional efficacy of *Ipomoea pes-caprae* and clarify its mechanism of action, we reconstructed the CIA model based on specific environments (rheumatic cold and heat), namely wind-dampness cold bi-syndrome and wind-dampness heat bi-syndrome models, based on the existing foundation [19,24,25]. Compared with the AIA model, wind-dampness cold bi-syndrome and wind-dampness heat bi-syndrome models can better simulate the pathogenesis of RA syndrome.

On the basis of the analysis of the chemical composition of the IPE, further analysis of its components in the body is of more important value and significance for the determination of the material basis of the medicinal effect and the study of the mechanism of action. In this study, we identified chemical components of 49 IPE based on the UPLC-MS technique, and eight absorbed components in plasma from the rats in the Con and WHM groups, and 10 absorbed components in plasma from the rats in the WCM group. One of the absorbed components in plasma, scopoletin, could target and regulate tyrosine kinases on fibroblast-like synoviocytes (FLS) to block NF-κB signaling, thereby attenuating the progression of RA [26]. Caffeic acid inhibits IL-6 and TNF-α and attenuates the inflammatory response in RA-FLS by blocking the phosphorylation of IκB and IκB kinases [27]. Ferulic acid inhibits JAK/STAT pathway-mediated RA by modulating the levels of C-reactive protein (CRP), rheumatoid factor (RF), TNF-α, and TGF-β, and increasing JAK2 levels [28]. Protocatechuic acid inhibits the secretion of MMP-3, MMP-13, TNF-α, IL-1β, and IL-6; regulates the expression of p-p65 and IκBα; and reduces the phosphorylation level of Akt and mTOR, and exerts anti-RA effects by inhibiting the NF-κB and Akt/mTOR signaling pathways [29]. Esculetin modulates RA by decreasing LTB4 levels [30]. It also exerts its anti-inflammatory effects by decreasing the secretion of NO and soluble intercellular adhesion molecules [31].

Next, we performed a serum metabolomics study in IPE to search for biomarkers and speculate on their metabolic pathways. It was found that a total of 11 potential biomarkers were screened after IPE intervention in WHM rats, of which 10 biomarkers were significantly up-regulated and one biomarker was significantly down-regulated. These biomarkers could mainly affect the metabolic pathways of histidine, phenylalanine, and tyrosine. A total of 10 potential biomarkers were screened after IPE intervention in WCM rats, of which three biomarkers were significantly up-regulated and seven biomarkers significantly down-regulated. These biomarkers mainly affected the metabolic pathways of histidine metabolism, fatty acid biosynthesis, alanine, aspartic acid, and glutamic acid metabolism. Compared with the WHM group, the serum levels of D-allose, D-mannitol, equine uric acid, L-threonate, pentachlorophenol, urocanic acid, creatinine, pyrocatechol, DL-norepinephrine, and malonic acid were up-regulated, while the content of methylhistidine was down-regulated. It is suggested that IPE may regulate the energy metabolism disorder of WHM rats in terms of amino acid metabolism and pyrimidine metabolism. Compared with the WCM group, the serum levels of urocanic acid, p-coumaryl alcohol, and D-mannose were up-regulated, while the levels of octanoic acid, capric acid, O-succinyl-L-homoserine, uracil, γ-aminobutyric acid, 2′-deoxycytidine, and cytosine were down-regulated in the WCM + IPE group, indicating that IPE might regulate the energy metabolism disorder in WCM rats in terms of amino acid metabolism, pyrimidine metabolism, and fatty acid metabolism.

In conclusion, IPE can adjust the content of biomarkers and make them return to normal levels, which has an obvious corrective effect, indicating that IPE can improve the metabolic profile abnormality and material metabolism disorder caused by wind-dampness heat bi-syndrome and wind-dampness cold bi-syndrome. This is one of the mechanisms of IPE’s anti-RA action.

We further carried out network pharmacological analysis to obtain four core targets and used KEGG to obtain two key signaling pathways associated with RA disease. ALB, also known as albumin (ALB), is the most important protein in human plasma [32]. The albumin-to-fibrinogen ratio (AFR) and C-reactive protein-to-albumin ratio (CAR) have become commonly used biomarkers to predict systemic inflammation [33]. High C-reactive protein (CRP) levels and low albumin (ALB) levels may be associated with chronic and severe inflammation. Several studies have shown that the CRP to ALB ratio (CAR) is associated with inflammatory status [34,35]. Recently, significantly increased fibrinogen (Fib) levels and lower ALB levels have been reported in RA patients [36,37]. AKT is protein kinase B, also known as PKB or Rac, and AKT1 is the main member of the AKT family. As a downstream molecule of the PI3K/Akt signaling pathway, when activated, AKT1 can inhibit cell proliferation and apoptosis, thus promoting cell survival. The PI3K/Akt signaling pathway could regulate the release and proliferation of inflammatory factors, apoptosis, and the formation of inflammation-related enzymes, and participate in the development of RA. The PI3K/Akt signaling pathway was found to be widespread and abnormally activated in RA synoviocytes [38]. Inhibition of the expression of the PI3K/Akt signaling pathway could induce apoptosis of fibroblast-like synoviocytes and have therapeutic effects on RA [39]. Phosphorylation of PI3K/Akt could also activate IL-1β and induce the expression of pro-inflammatory factors TNF-α and IL-6 and joint inflammation in rats [40]. EGFR (epidermal growth factor receptor, EGFR) is a member of the epidermal growth factor receptor (HER) family, and binding of EGFR to its corresponding ligands could activate several intracellular signaling pathways, such as the PI3K/Akt and MAPK pathways. EGFR and its ligands can also induce cytokine Caspase 3 (CASP3) production in synovial fibroblasts during the pathogenesis of RA [41]. It has been found that in RA patients, TNF induces caspase 3/GSDME activation and triggers monocyte and macrophage pyroptosis [42]. Therefore, inhibition of TNF-induced caspase 3/GSDME-mediated pyroptosis attenuates RA. IL-17 is a pro-inflammatory cytokine that promotes stromal cell proliferation, osteoclast differentiation, and angiogenesis in RA. IL-17 has been shown to activate many common downstream signaling pathways, including NF-κB, MAPKs, JNK, p38, ERK, C/EBP, PI3K, and JAK/STATs. Prolonged intra-articular administration of IL-17 in mice leads to the emergence of key features of RA, such as inflammation and destruction of articular bone and cartilage [43]. IL-17 can also cause bone destruction by inducing osteoclastogenesis [44]. In addition, upregulation of TNF and IL-17 can lead to further stimulation of fibroblasts and epithelial cells to secrete IL-6, IL-8, PGE2, and neutrophil chemotactic agents, thereby exacerbating the development of inflammation [45].

Finally, we combined metabolomics and network pharmacology analyses to identify the key targets of IPE for RA as ALB and AKT1, with endogenous biomarkers being D-mannose and cytosine, and the key signaling pathways involved are SLC-mediated transmembrane transport and C-type lectin receptor signaling pathway. In addition, we also found that the four absorbed components in plasma, namely, scopoletin, methyl caffeate, protocatechuic acid, and ferulic acid, had strong binding capacities with ALB and AKT1, respectively, through molecular docking validation, suggesting that scopoletin, caffeic acid methyl ester, protocatechuic acid, and ferulic acid of IPE may exert anti-RA effects by regulating ALB and AKT1.

## 4. Materials and Methods

### 4.1. Chemical Reagents and Standards

Methanol, acetonitrile, and formic acid for mass spectrometry were purchased from Thermo Fisher Scientific (168 Third Avenue, Waltham, MA 02451, USA.); natural bovine collagen type II was purchased from Merck (Frankfurter Straße 250, 64293 Darmstadt Germany); complete Freund’s adjuvant was purchased from SIGMA (3050 Spruce Street St., Saint Louis, MO, USA); Duhuo formula granules and Fangji formula granules were purchased from PuraPharm (No. 9, Jinhe Road Nanning Economic and Technological Development Area, Nanning, China) Corporation; and IL-6 and TNF-α were purchased from Elabscience Biotechnology Co., Ltd. (No. 6, Jiufu Road, Jiangxia District, Wuhan, China). Caffeic acid (MUST-22062118), scopoletin (MUST-21032415), protocatechuic acid (MUST-22041413), syringic acid (MUST-22052411), and ferulic acid (MUST-22052411) were purchased from Shanghai Acmec Biochemical Technology Co., Ltd. (No. 2991, Jinke Road, Pudong New District, Shanghai, China). The purity of the above controls was ≥98%.

### 4.2. Preparation of Plant Extracts

#### 4.2.1. Preparation of IPE Paste

*Ipomoea pes-caprae* medicinal materials were collected from Baisha Town, Hepu County, Beihai City, Guangxi Province, on 8 April 2022, and were identified as the above-ground part of *Ipomoea pes-caprae* (L.) Sweet of the family Convolvulaceae by Professor Songji Wang of Guangxi University of Chinese Medicine. The IPE was prepared in the Guangxi Key Laboratory of Efficacy Study on Chinese Materia Medica. Briefly, the above-ground part of *Ipomoea pes-caprae* was taken, 12 times the amount of water was added, soaked for 1 h, heated to boiling, extracted for 1 h, and filtered while hot. The above steps were repeated twice, and the two filtrates were combined, concentrated under reduced pressure, and stored at −20 °C for future use.

#### 4.2.2. Preparation of IPE Samples

The IPE paste obtained under item 2.2.1 was dried to powder, and its powder of 1.0 g was accurately weighed. The weighed powder was mixed with 20 mL of 50% methanol and subjected to ultrasonic extraction for 30 min. After cooling to room temperature, the volume was adjusted to the initial weight with 50% methanol. The solution was diluted to a final concentration of 1 mg/mL, filtered through a 0.22 μm membrane, and 3 μL of the filtrate was injected for UPLC-MS analysis.

### 4.3. Animal Grouping and Drug Administration

A total of 83 male SD rats, SPF grade, six weeks old, body mass (200 ± 10 g), were purchased from Hunan SJA Laboratory Animal Co., Ltd. (Haodan Science and Technology Park, No.99 Changchong Road, Longping High-tech Park, Furong District, Changsha City, China), Animal License No. SCXK (Hunan) 2019-0004. The animal experiments were approved by the Animal Ethics Committee of Guangxi University of Chinese Medicine (Approval No. DW20220220-117). After one week of adaptive feeding, the animals were randomly divided into eight groups of 10 animals according to weight stratification: normal control group (Con), natural bovine type II collagen model group (CII), wind-dampness cold bi-syndrome model group (WCM), wind-dampness heat bi-syndrome model group (WHM), wind-dampness cold bi-syndrome + *Ipomoea pes-caprae* group (WCM + IPE), wind-dampness heat bi-syndrome + *Ipomoea pes-caprae* group (WHM + IPE), wind-dampness cold bi-syndrome + Duhuo group (WCM + DH) (Duhuo formula granules), and wind-dampness heat bi-syndrome + Fangji group (WHM + FJ) (Fangji formula granules). Another three rats were randomly selected as the normal administration group (Con + IPE), and the absorbed components in plasma were studied in vivo. The oral dose of IPE was 13.5 g/kg, and the dosing volume was 20 mL/kg. The oral doses of Duhuo formula granules and Fangji formula granules were 9.0 g/kg, with a dosing volume of 20 mL/kg for each. The blank control group and each model group were administered an equal volume of distilled water by oral gavage. The administration was performed once daily for 28 consecutive days.

### 4.4. Animal Model Construction

**Figure 12:**
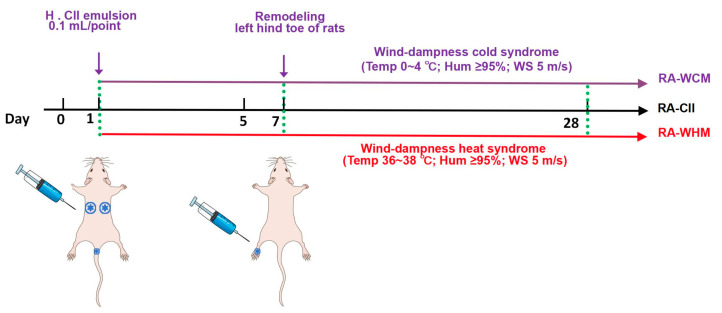
The process diagram of animal model establishment.

A solution of natural bovine type II collagen (CII) was mixed with freund’s complete adjuvant (CFA) in equal volumes (1:1; *v*:*v*) to form a CII emulsion. On the first day of the experiment, except for the normal control group, rats in all other groups were subcutaneously injected with a total of 0.3 mL of CII emulsion, divided into two injection sites on the back (on either side of the spine) and one site at the base of the tail, with 0.1 mL of CII emulsion at each site. On the seventh day of the experiment, a second modeling was performed, and 0.1 mL of CII emulsion was injected subcutaneously into the left hind toe of the rat [18]. After the first injection of CII emulsion, the rats were placed in an environment that simulated wind-dampness cold bi-syndrome (temperature 0–4 °C, relative humidity ≥ 95%, wind speed 5 m/s), once a day, 30 min each time, for a total of 28 days. On the contrary, after the first injection of CII emulsion, the rats were placed in an environment that simulated wind-dampness heat bi-syndrome (temperature 36–38 °C, relative humidity ≥ 95%, wind speed 5 m/s), once a day for 30 min each time, for a total of 28 days. A schematic diagram of the animal model is shown in Figure 12.

### 4.5. Joint Inflammation AI Score Evaluation and Left Hind Toe Swelling Analysis

During the experiment, we systematically observed the lesions of the left hind toe and other toes of rats in each group according to the following time nodes: Day 7, Day 14, Day 21, Day 28, Day 35, Day 42, Day 49, and Day 56, and used the AI scoring system to evaluate them. The specific criteria for the AI score are as follows: 0 points: normal; 1 point: slight but obvious redness and swelling of the ankle joint, obvious redness and swelling limited to individual toe joints; 2 points: moderate redness and swelling of the toe joints; 3 points: severe redness and swelling of the entire paw, including the toes; 4 points: the toes are inflamed to the greatest extent, involving multiple joints. The sum of the scores for the four paws of each rat is taken as the final arthritis score, with a maximum of 16 points. AI score ≥ 4 was used as a reference criterion for successful replication of the RA model [46].

In addition, the thickness of the left hind toe of rats was measured using a vernier caliper every seven days to calculate the degree of toe swelling degree. The degree of swelling = (C_t_ − C_0_)/C_0_, where C_0_ represents the plantar thickness before modeling, and C_t_ indicates the toe thickness after modeling.

### 4.6. Serum Sample Collection

Rats were fasted for 12 h before administration but not deprived of water, then weighed and administered. A total of 0.2 mL of blood was collected from the orbit at 5 min, 30 min, 45 min, 1 h, 2 h, 4 h, 6 h, 8 h, and 12 h after the end of gavage administration and placed in a centrifuge tube with a preservative anticoagulant (1.5% EDTA aqueous solution, 1.5 mL). The samples were centrifuged at 4 °C for 10 min at 4000 rpm to separate the plasma. The supernatant was transferred to a new centrifuge tube and stored in a refrigerator at −80 °C for the detection of absorbed components in plasma and pharmacokinetics. In addition, after the end of the entire experimental cycle, blood was taken from the abdominal aorta of the rat. The blood samples were kept at room temperature for 2 h, then centrifuged at 4 °C at 3000 rpm for 10 min, and the upper serum was aspirated. The samples were stored at −80 °C in a low-temperature refrigerator for TNF-α, IL-6 detection, and metabolomics detection.

QC samples were prepared by taking equal amounts of all samples to be tested and mixing them into the same EP tube. Pipette 1 mL of serum from QC and each of the 8 groups (Con, CII, WCM, WHM, WCM + IPE, WHM + IPE, WCM + DH, and WHM + FJ) and add three times the volume of methanol and acetonitrile solution (methanol: acetonitrile = 1:1) to precipitate the protein. Vortex for 3 min, then centrifuge at low temperature for 10 min (15,000 r/min, 4 °C); the supernatant was passed through a 0.22 μm microporous filter membrane, dried to a solid state under nitrogen, and 200 μL of methanol was added to the pellet. The mixture was sonicated for 3 min, mixed by vortexing for 1 min, centrifuged at low temperature for 10 min (15,000 r/min, 4 °C), and the supernatant was transferred for UPLC-MS analysis.

### 4.7. Histopathology and Micro-CT Analysis

The left hind-toe ankle joint of the rat obtained in the experiment was stripped of the surface skin and muscles and placed in a 4% paraformaldehyde solution for dehydration and fixation. After one day, it was replaced with a new 4% paraformaldehyde solution for continued fixation for seven days. After dehydration and fixation, the ankle joint was decalcified in EDTA decalcification solution for four weeks. The tissue was dehydrated, embedded in paraffin, sectioned, and the sections were dewaxed before being stained with HE and analyzed by micro-CT. The scanning parameters for the micro-CT imaging system were as follows: X-rays, 90 kV and 80 μA; field of view, 18 mm; voxel size, 36 μm; scan mode, high resolution; scan time, 14 min. Scan-reconstructed images were analyzed using Analyze 12.0 software.

### 4.8. Chromatographic and Mass Spectrometry Conditions for UPLC-MS

Ultra-high liquid chromatograph (model: ExionLC AC, manufacturer: SCIEX Corporation, USA) and QTOF mass spectrometer (model: X500R, manufacturer: SCIEX Corporation, USA), Waters ACQUITY UPLC HSS T3 C18 column (1.8 μm, 2.1 × 100 mm) was used as the mobile phase for the separation of 0.1% formic acid aqueous solution (A)-acetonitrile (B) as a mobile phase, gradient elution (0~30 min, 5%~95% B); volume flow rate of 0.4 mL/min; injection volume: 3 μL, column temperature: 40 °C. Ion source: dual-spray TurboV, positive and negative ion scanning, TOF MS scanning range of *m/z* 100~1500, positive/negative ion source voltage of −4500 V/5500 V, ion source temperature: 600 °C, GS1: 55 psi, GS2: 55 psi, air curtain gas pressure: 35 psi, parent ion collision energy: ±10 V, de-cluster voltage (DP): ±80 V, daughter ion collision energy (CE): ±35 V.

### 4.9. Pharmacokinetic Analysis

In order to further elaborate on the in vivo pharmacokinetic characteristics of IPE, this study selected caffeic acid, protocatechuic acid, and ferulic acid as the research objects to explore their in vivo absorption and metabolism characteristics. In addition, syringic acid was chosen as the internal standard. The column is the same as under “2.8”. Use A as 0.1% acetic acid aqueous solution (A) acetonitrile (B) as the mobile phase, with gradient elution (0–6 min, 5–20% B; 6–10 min, 20–40% B), a volume flow of 0.4 mL/min, a column temperature of 40 °C, and an injection volume of 3 μL. Ion source: electrospray ion source (ESI), negative ion mode collection, simultaneous measurement by multiple reaction monitoring (MRM) mode. The MS/MS setting parameters were as follows: atomizer pressure was 35 psi, drying air temperature was 150 °C, capillary voltage was 4500 V, and sheath air temperature was 600 °C. The ion pairs used for quantitative analysis are shown in Table 8.

### 4.10. Serum Metabolomics Analysis

In order to elucidate the changes in endogenous components observed in model rats after IPE administration, this study used serum metabolomics technology to compare the differential metabolites before and after IPE administration, screen biomarkers, and predict metabolic pathways. The UPLC conditions were water + 25 mM ammonium acetate + 25 mM ammonia (A)-acetonitrile (B) as the mobile phase, with a gradient elution program (0–1.5 min, 98–98% B; 1.5–12 min, 98–2% B; 12–14 min, 2–2% B; 14–14.1 min, 2–98% B; 14.1–17 min, 98–98% B). The flow rate was 0.3 mL/min, the injection volume was 2 μL, and the column temperature was 25 °C. The conditions of mass spectrometry were as follows: ion source: electrospray ionization (ESI); acquisition mode: positive ion and negative ion mode; ion source temperature: 600 °C; nebulizing gas auxiliary heating gas 1: nitrogen, 60 psi; auxiliary heating gas 2: nitrogen, 60 psi; air curtain gas, 30 psi; spray voltage: ±5500 V; first-stage scanning range: 80–1200 Da; resolution: 60,000. Scan accumulation time: 100 ms; the second level adopts the segmented acquisition method; the scanning range: 70–1200 Da; the second level resolution: 30,000; scan accumulation time: 50 ms; dynamic exclusion time: 4 s. To ensure the stability of the method, QC samples were tested on the machine before, during and after the injection of the samples to be tested.

### 4.11. Multivariate Data Analysis

The raw data were collected using the UPLC-MS method, and peak alignment, retention time correction, and peak area extraction were performed on the raw data. The peak area-related parameters were extracted as follows: centWave: *m*/*z* 10 ppm; peak width: c (10, 60); prefilter: c (10, 100). Peak grouping parameters, bw = 5, mzwid = 0.025, minfrac = 0.5. Annotation of isotopes and adducts was performed using CAMERA (Collection of Algorithms for MEtabolite pRofile Annotation). Metabolite identification was based on the mass-to-charge ratio (*m*/*z* < 10 ppm), secondary profiles, and a database established with standards. PCA was utilized to observe natural clustering trends and metabolic pattern differences among each group, followed by Orthogonal partial least squares discriminant analysis (OPLS-DA) to identify differences between the two groups. According to the mass-to-charge ratio of the components, the screening condition: Mass Error < 5 ppm, combined with secondary spectral information. The possible structural types of the differential metabolites were hypothesized, and the online databases PubChem (https://pubchem.ncbi.nlm.nih.gov) (accessed on 2 March 2023) and HMDB (https://hmdb.ca) (accessed on 2 March 2023) were searched to determine their compositions.

### 4.12. Network Pharmacology Analysis

Potential targets of the absorbed components of IPE in the plasma were obtained from the Pharm Mapper (https://www.lilab-ecust.cn/pharmmapper/) (accessed on 21 May 2024) and TCMSP (https://old.tcmsp-e.com/tcmsp.php) (accessed on 21 May 2024). The disease targets for wind-dampness cold bi-syndrome and wind-dampness heat bi-syndrome were retrieved from OMIM (https://www.omim.org/) (accessed on 21 May 2024) and Gene Cards (https://www.genecards.org/) (accessed on 21 May 2024) databases using “rheumatoid arthritis” as the search term. The target protein names were corrected from UniProt (https://www.uniprot.org/) (accessed on 22 May 2024) to the official gene names. Venny diagrams were produced by Venny 2.1.0 (https://bioinfogp.cnb.csic.es/tools/venny/index.html) (accessed on 26 May 2024), and the incoming component targets were intersected with disease targets, and the intersecting targets were potential therapeutic targets of IPE in RA. Gene Ontology (GO) enrichment and Kyoto Encyclopedia of Genes and Genomes (KEGG) pathway enrichment analyses of the intersecting targets were performed using the DAVID database (https://david.ncifcrf.gov/) (accessed on 29 May 2024) to explore the biological functions and potential mechanisms of the detected targets. The obtained target genes were submitted to the interaction gene search tool STRING (https://cn.string-db.org/) (accessed on 15 June 2024) to construct protein–protein interaction (PPI) networks. The PPI network data were imported into Cytoscape 3.10 software to construct the “absorbed components-target-disease” network of IPE for the treatment of wind-dampness cold bi-syndrome and wind-dampness heat bi-syndrome. The endogenous biomarkers identified by metabolomics and the targets obtained from the network pharmacology intersection were imported into Integrated Molecular Pathway Level Analysis (IMPaLA, http://impala.molgen.mpg.de/impala) (accessed on 20 July 2024) and Cytoscape 3.10 software to construct the biomarker-pathway-target visualization network.

### 4.13. Molecular Docking

The 3D structures of the absorbed components in plasma were downloaded from the TCMSP and PubChem databases and converted to mol2 format files using Open Babel 3.1.1. The core targets with the top-ranked degree value in the PPI network were selected, and the 3D structure of the desired protein was downloaded by searching the PDB database and saved in pdb format. AutoDockTools-1.5.7 software was used to molecularly dock the absorbed components in plasma and core target proteins, and finally, PyMOL 2.5.2 software was used to visualize the results with higher activity.

### 4.14. Statistical Methods

Expressed as mean ± standard deviation ($\bar{x}$ ± *s*) and processed by GraphPad Prism 8.4.3 statistical software, statistical comparisons were evaluated by one-way ANOVA, followed by the Student–Newman–Keuls post hoc test. The difference was statistically significant with *p* < 0.05 and *p* < 0.01. The peak area of each individual compound extracted from IPE was compared to the sum of the peak areas of the total compounds of IPE, and the relative content of each component in the total compounds was calculated based on the ion flow peak area normalization method [47]. VIP > 1 and *p*-value < 0.05 were used to screen significantly changed metabolites. Pearson correlation analysis was conducted to determine the correlation between the two variables.

## 5. Conclusions

This experiment proves that IPE can effectively alleviate RA symptoms, and its multiple chemical components are well absorbed and metabolized in vivo, while also exploring the key targets and mechanisms of IPE in the treatment of RA based on serum metabolomics and network pharmacology. The results showed that IPE exhibited significant therapeutic effects on both wind-dampness cold bi-syndrome and wind-dampness heat bi-syndrome by acting on multiple potential targets and metabolic pathways, especially the core targets of ALB and AKT1. This study provides data and theoretical support for the in-depth investigation of the mechanism of IPE against RA and lays the foundation for the clinical application of IPE.

## Figures and Tables

**Table 1:** Comparison of AI score in rats in each group ($\bar{x}$ ± *s*, *n* = 10.)

Group	Dose (g/kg)	AI Score	
		Day 28 (Prior to First Dose)	Day 56 (Post Last Dose)
Con	-	0 ± 0	0 ± 0
CII	-	6.4 ± 0.8 **	7.4 ± 2.8 **
WCM	-	5.9 ± 0.9 **	7.5 ± 3.6 **
WHM	-	6.3 ± 1.3 **	7.4 ± 1.8 **
WCM + DH	9	6.4 ± 1.3 **	4.9 ± 0.9 ^Δ^
WHM + FJ	9	6.9 ± 0.7 **	3.6 ± 1.2 ^#^
WCM + IPE	13.5	5.8 ± 1.4 **	4.0 ± 0.8 ^Δ^
WHM + IPE	13.5	6.4 ± 1.3 **	3.4 ± 0.8 ^#^

The data were analyzed via one-way analysis of variance (ANOVA) followed by the Student–Newman–Keuls post hoc test. ** *p* < 0.01, significantly different from the Con; ^Δ^ *p* < 0.05, significantly different from the WCM; ^#^ *p* < 0.05, significantly different from the WHM.

**Table 2:** Comparison of left hind toes swelling in rats in each group ($\bar{x}$ ± *s*, *n* = 10.)

Group	Dose (g/kg)	left Hind Toes Swelling	
		Day 28 (Prior to First Dose)	Day 56 (Post Last Dose)
Con	-	0.13 ± 0.03	0.07 ± 0.03
CII	-	0.44 ± 0.16 **	0.47 ± 0.13 **
WCM	-	0.50 ± 0.31 **	0.58 ± 0.22 **
WHM	-	0.40 ± 0.18 **	0.53 ± 0.14 **
WCM + DH	9	0.38 ± 0.24 **	0.29 ± 0.06 ^Δ^
WHM + FJ	9	0.35 ± 0.07 **	0.28 ± 0.09 ^#^
WCM + IPE	13.5	0.40 ± 0.15 **	0.25 ± 0.03 ^Δ^
WHM + IPE	13.5	0.37 ± 0.11 **	0.29 ± 0.04 ^#^

The data were analyzed by one-way analysis of variance (ANOVA) followed by the Student–Newman–Keuls post hoc test. ** *p* < 0.01, significantly different from the Con; ^Δ^ *p* < 0.05, significantly different from the WCM; ^#^ *p* < 0.05, significantly different from the WHM.

**Table 3:** The identified chemical composition of IPE based on the UPLC-MS method.

Peak No.	Formula	Retention Time (min)	Fragment Ion	Relative Molecular Mass (*m*/*z*)		Error (ppm) ×10^6^	Fragment Ions in the Positive/Negative ion Mode (*m*/*z*)	Identification	Relative Content (%)	Identification Type
				Precursor Mass	Found at Mass					
1	C_7_H_12_O_6_	0.53	[M−H]^−^	191.0561	191.0562	0.4	173.0459, 127.0402, 109.0297	Quinic acid	1.918	Organic acid
2	C_4_H_4_O_4_	0.59	[M−H]^−^	115.0036	115.0037	0.3	97.9325, 71.0130	Fumaric acid	0.129	Organic acid
3	C_4_H_6_O_5_	0.6	[M−H]^−^	133.0143	133.0144	1.1	71.0139, 72.9931, 59.0138, 115.0039	Malic acid	0.626	Organic acid
4	C_6_H_8_O_7_	0.72	[M−H]−	191.0197	191.0200	1.4	129.0198, 111.0091	Citric acid	1.839	Organic acid
5	C_10_H_13_N_5_O_4_	0.76	[M+H]^+^	268.1040	268.1039	−0.5	136.0615, 119.0351	Adenosine	0.441	Nucleoside
6	C_4_H_6_O_4_	0.79	[M−H]^−^	117.0193	117.0193	0	117.0193, 99.9260, 73.0294	Succinic acid	0.034	Organic acid
7	C_10_H_13_N_5_O_5_	0.8	[M−H]^−^	282.0844	282.0846	0.8	150.0422, 133.0156	Guanosine	0.104	Adenosine
8	C_7_H_6_O_5_	1.03	[M−H]^−^	169.0143	169.0141	−0.7	79.0185, 125.0250, 124.0251, 69.0338, 51.0242	Gallic acid	0.015	Organic acid
9	C_7_H_6_O_4_	1.75	[M−H]^−^	153.0193	153.0195	1.3	108.0221, 109.0290	Protocatechuic acid	0.296	Organic acid
10	C_16_H_18_O_9_	2.03	[M−H]^−^	353.0878	353.0880	0.6	191.0579, 179.0347, 135.0453, 161.0245	Neochlorogenic acid	11.241	Organic acid
11	C_7_H_6_O_3_	2.36	[M−H]^−^	137.0244	137.0245	0.1	137.0245, 119.0140, 109.0296, 108.0219, 93.0343	Protocatechuic aldehyde	1.34	Organic acid
12	C_7_H_6_O_3_	2.36	[M−H]^−^	137.0244	137.0245	0.1	137.0245, 93.0343, 65.0033	Salicylic acid	1.34	Organic acid
13	C_15_H_16_O_9_	2.38	[M−H]^−^	339.0722	339.0721	−0.2	177.0192, 133.0295	Esculin	0.041	Coumarins
14	C_8_H_8_O_3_	2.97	[M−H]^−^	151.0401	151.0400	−0.5	136.0168, 108.0216	Methyl 4-hydroxybenzoate	0.043	Organic acid
15	C_16_H_18_O_9_	3.03	[M−H]^−^	353.0878	353.0879	0.2	191.0584, 173.0455, 135.0450, 179.0350, 161.0246	Chlorogenic acid	15.739	Organic acid
16	C_16_H_18_O_9_	3.03	[M−H]^−^	353.0878	353.0879	0.2	1910561, 179.0349, 135.0451, 161.0241	Cryptochlorogenic acid	2.335	Organic acid
17	C_10_H_8_O_4_	3.08	[M+H]^+^	193.0495	193.0494	−0.7	178.0262, 150,0313, 133.0285, 122.0363	Scopoletin	0.575	Coumarins
18	C_9_H_10_O_2_	3.11	[M−H]^−^	149.0608	149.0606	−1.1	149.0581, 121.0688	4-Hydroxy-3-methoxystyrene	0.021	Organic acid
19	C_10_H_10_O_4_	3.13	[M−H]^−^	193.0506	195.0504	−1	134.0360, 133.0270	Ferulic acid	0.14	Organic acid
20	C_9_H_6_O_4_	3.18	[M+H]^+^	179.0339	179.0337	−1	151.0398, 123.0443,	Esculetin	0.053	Coumarins
21	C_9_H_8_O_4_	3.25	[M−H]^−^	179.0350	179.0349	−0.3	135.0453, 134.0375, 107.0504, 89.0398, 79.0554	Caffeic acid	3.633	Organic acid
22	C_17_H_20_N_4_O_6_	3.89	[M+H]^−^	377.1456	377.1455	−0.1	377.1427, 243.0877, 172.0873, 319.1363	Vitamine B2	0.201	Vitamins
23	C_9_H_6_O_2_	4.51	[M+H]^+^	147.0441	147.0441	0	119.0487	Coumarin	0.097	Coumarins
24	C_27_H_30_O_17_	4.73	[M−H]^−^	625.1410	625.1410	0	625.1413, 463.1073, 301.0361, 271.0254	Quercetin-3-O-sophoroside	0.095	Flavonoids
25	C_10_H_10_O_3_	4.91	[M+H]^−^	179.0703	179.0609	−1.9	133.1009, 105.0700	Mellein	0.029	Organic acid
26	C_10_H_8_O_4_	4.98	[M+H]^−^	193.0495	193.0492	−1.8	178.0259, 150.0310, 133.0284, 122.0362	Scopoletin	0.787	Coumarins
27	C_10_H_10_O_4_	5.11	[M−H]^−^	193.0506	193.0503	−1.4	133.0285, 134.0361	Methyl caffeate acid	0.042	Organic acid
28	C_27_H_30_O_16_	5.39	[M−H]^−^	609.1461	609.1458	−0.5	609.1455, 301.0349, 300.0273, 271.0245	Rutin	0.155	Flavonoids
29	C_21_H_20_O_12_	5.59	[M−H]^−^	463.0882	463.0883	0.2	301.0354, 300.0276, 271.0249, 255.0300	Isoquercitrin	0.762	Flavonoids
30	C_21_H_20_O_12_	5.71	[M−H]^−^	463.0882	463.0881	−0.1	301.0353, 300.0275, 271.0249, 255.0299	Quercetin-7-O-β-D-glucopyranoside	2.043	Flavonoids
31	C_7_H_6_O_3_	5.75	[M−H]^−^	137.0244	137.0242	−1.8	93.0348, 65.0396	p-Hydroxybenzoic acid	0.074	Organic acid
32	C_27_H_30_O_15_	5.88	[M−H]^−^	593.1512	593.1511	−0.3	284.0331	Kaempferol-3-O-rutinoside	0.026	Flavonoids
33	C_25_H_24_O_12_	6.09	[M−H]^−^	515.1195	515.1194	−0.2	353.0875, 191.0557, 179.5898, 173.0475	Isochlorogenic acid B	19.91	Organic acid
34	C_11_H_12_O_4_	6.138	[M+H]^+^	209.0808	209.0808	0	103.0544	Ethyl Caffeate	0.074	Organic acid
35	C_15_H_10_O_6_	6.15	[M+H]^+^	287.0550	287.0540	−3.5	153.0185, 121.0291	Kaempferol	0.147	Flavonoids
36	C_21_H_20_O_11_	6.15	[M−H]^−^	447.0933	447.0929	−1	284.0327, 255.0295, 227.0346	Astragalin	0.123	Flavonoids
37	C_26_H_32_O_11_	6.16	[M−H]^−^	519.1872	519.1871	−0.4	357.1344, 151.0402, 136.0165	(+)-Pinoresinol-β-D-glucoside	0.108	Flavonoids
38	C_25_H_24_O_12_	6.31	[M−H]^−^	515.1195	515.1192	−0.6	353.0875, 191.6309, 179.0347, 173.0457	Isochlorogenic acid A	12.564	Organic acid
39	C_22_H_26_O_8_	6.41	[M−H]^−^	417.1555	417.1550	−1.2	387.1084, 181.0503, 166.0269, 137.0242	Syringaresinol	0.167	Organic acid
40	C_25_H_24_O_12_	6.75	[M−H]^−^	515.1195	515.1191	−0.8	353.0873, 191.0555, 179.0357, 173.0480	Isochlorogenic acid C	19.627	Organic acid
41	C_26_H_26_O_12_	8.01	[M−H]^−^	529.1352	529.1348	−0.7	367.1033, 353,0870, 179.0350, 173.0454	3,5-Di-caffeoylquinic acid or 4,5-Di -caffeoylquinicacidmethyl Ester or 3,4-Di- caffeoylquinic acid methyl ester	0.302	Organic acid
42	C_34_H_30_O_14_	8.26	[M−H]^−^	661.1563	661.1561	−0.3	515.1193, 499.1245, 353.0880, 191.0558, 179.0349	3,5-Di-O-caffeoyl-4-O-coumaroylquinic acid	0.27	Organic acid
43	C_18_H_16_O_6_	9.53	[M+H]^+^	329.1020	329.1021	0.5	314.0787, 299.0552	4′-Hydroxy-3′,5,7, -trimethoxyflavone	0.242	Flavonoids
44	C_36_H_58_O_10_	10.27	[M+FA−H]^−^	695.4012	695.4005	−1.0	487.3421	Pedunculoside	0.102	Terpene
45	C_43_H_36_O_16_	10.93	[M−H]^−^	807.1931	807.1925	−0.8	645.1607, 499.1238, 179.0354	4,5-Di-O-caffeoyl-1,3-Di-O-couma-roylquinicacid	0.021	Organic acid
46	C_42_H_62_O_16_	11.84	[M−H]^−^	821.3965	821.3968	0.3	821.3948, 351.0563	Glycyrrhizic Acid	0.009	Organic acid
47	C_13_H_14_O_2_	17.76	[M+H]^+^	203.1067	203.1066	−0.4	185.0957, 161.0960, 121.0653	2-Hydroxy-4,4,7-trimethyl-1(4H)-naphthalenone	0.086	Organic acid
48	C_61_H_106_O_24_	20.97	[M+FA−H]^−^	1267.7056	1267.7057	0.2	965.4911, 947.4778, 545.3325, 417.2858, 146.9649	Pescapreins XXX	0.001	Resin glycosides
49	C_18_H_34_O_2_	24.36	[M−H]^−^	281.2481	281.2479	−2.5	281.7474	Oleic acid	0.032	Organic acid

**Table 4:** Identification of absorbed components in plasma of IPE in rats of each administration group.

Group	No.	Retention Time (min)	Formula	Fragment Ion	Relative Molecular Mass (*m*/*z*)		Error (ppm) ×10^6^	Fragment Ions in the Positive/Negative Ion Mode (*m*/*z*)	Identification	Relative Content (%)	>Identification Type
					Precursor Mass	Found at Mass					
Con + IPE	1	1.98	C_7_H_6_O_4_	[M−H]^−^	153.0193	153.0197	2.5	109.0305, 108.0225	Protocatechuic acid	1.04	Prototypical or metabolic components
	2	3.26	C_10_H_8_O_4_	[M+H]+	193.0495	193.0495	0	178.0269, 150.0323, 133.0297, 122.0378	Scopoletin	5.83	Prototype components
	3	3.34	C_9_H_8_O_4_	[M−H]^−^	179.035	179.0353	1.6	135.0453, 117.0340, 89.0391	Caffeic acid	33.04	Prototypical or metabolic components
	4	3.42	C_9_H_6_O_4_	[M+H]+	179.0339	179.0343	2.0	123.0455	Esculetine	0.39	Prototype components
	5	3.6	C_10_H_10_O_4_	[M−H]^−^	193.0506	193.0507	0.3	134.0380, 133.0296	Ferulic acid	17.23	Prototypical or metabolic components
	6	5.22	C_10_H_8_O_4_	[M+H]+	193.0495	193.0493	−1.0	178.0270, 150.0317, 133.0299, 122.0378	Isoscopoletin	2.7	Prototype components
	7	5.39	C_10_H_10_O_4_	[M−H]^−^	193.0506	193.0511	2.6	134.0373, 133.0289	Methyl caffeate acid	1.57	Prototypical or metabolic components
	8	6.05	C_7_H_6_O_3_	[M−H]^−^	137.0244	137.0242	−1.2	93.0334, 65.0391	p-Hydroxybenzoic acid	38.2	Prototypical or metabolic components
WCM + IPE	9	1.75	C_7_H_6_O_4_	[M−H]^−^	153.0193	153.0196	2	109.0291, 108.0207	Protocatechuic acid	1.14	Prototypical or metabolic components
	10	2.15	C_7_H_6_O_3_	[M−H]^−^	137.0244	137.0246	1.6	108.0226, 93.0345	Protocatechuic aldehyde	1.68	Prototype components
	11	3.16	C_10_H_8_O_4_	[M+H]^+^	193.0495	193.0494	−0.1	178.0271, 150.0322, 133.0289, 122.0369	Scopoletin	4.71	Prototype components
	12	3.19	C_9_H_8_O_4_	[M−H]^−^	179.0350	179.0352	1.2	135.0454, 117.0346, 89.0400	Caffeic acid	30.2	Prototypical or metabolic components
	13	3.31	C_9_H_6_O_4_	[M+H]^+^	179.0339	179.0340	0.8	151.0398, 123.0442	Esculetine	0.5	Prototype components
	14	3.48	C_10_H_10_O_4_	[M−H]^−^	193.0506	193.0506	0	134.0363, 133.0291	Ferulic acid	20.09	Prototypical or metabolic components
	15	4.69	C_9_H_6_O_2_	[M+H]^+^	147.0441	147.0440	−0.7	119.0500	Coumarin	1.77	Prototype components
	16	5.18	C_10_H_8_O_4_	[M+H]^+^	193.0495	193.0486	−5	178.0270, 150.0321, 133.0291, 122.0366	Isoscopoletin	7.8	Prototype components
	17	5.32	C_10_H_10_O_4_	[M−H]^−^	193.0506	193.0505	0.6	133.0285, 134.0361	Methyl caffeate acid	2.45	Prototypical or metabolic components
	18	5.96	C_7_H_6_O_3_	[M−H]^−^	137.0244	137.0242	−1.3	93.0334, 65.0390	p-Hydroxybenzoic acid	29.65	Prototypical or metabolic components
WHM + IPE	19	1.79	C_7_H_6_O_4_	[M−H]^−^	153.0193	153.0192	−0.9	109.0308, 108.0219	Protocatechuic acid	0.84	Prototypical or metabolic components
	20	3.23	C_10_H_8_O_4_	[M+H]^+^	193.0495	193.0497	1	178.0280, 150.0334, 133.0299, 122.0369	Scopoletin	5.56	Prototype components
	21	3.32	C_9_H_8_O_4_	[M−H]^−^	179.0350	179.0349	−0.7	135.0454, 107.0509, 89.0391	Caffeic acid	34.24	Prototypical or metabolic components
	22	3.41	C_9_H_6_O_4_	[M+H]^+^	179.0339	179.0342	1.7	151.0141, 123.0439	Esculetine	0.58	Prototype components
	23	3.58	C_10_H_10_O_4_	[M−H]^−^	193.0506	193.0505	−0.6	134.0369, 133.0294	Ferulic acid	17.71	Prototypical or metabolic components
	24	5.21	C_10_H_8_O_4_	[M+H]^+^	193.0495	193.0489	−3.2	178.0259, 150.0310, 133.0284, 122.0368	Isoscopoletin	5	Prototype components
	25	5.38	C_10_H_10_O_4_	[M−H]^−^	193.0506	193.0508	1	134.0510, 133.0292	Methyl caffeate acid	2.02	Prototypical or metabolic components
	26	6.05	C_7_H_6_O_3_	[M−H]^−^	137.0244	137.0241	−2.1	93.0333, 65.0391	p-Hydroxybenzoic acid	34.04	Prototypical or metabolic components

**Table 5:** Pharmacokinetic parameters of the main components of the IPE in rats of each administration group in vivo (x ± s, *n* = 6).

	Parameters	AUC_0~t_ (μg·h·L^−1^)	MRT_0~t_ (h)	T_max_ (h)	C_max_ (μg·L^−1^)	t_1/2_ (h)
Group Component						
Con + IPE	Caffeic acid	2078.79 ± 349.44	4.18 ± 1.82	0.85 ± 0.22	702.53 ± 189.91	4.51 ± 3.03
	Ferulic acid	60.56 ± 27.27	1.30 ± 0.28	0.45 ± 0.10	35.76 ± 10.90	1.51 ± 0.51
	Protocatechuic acid	504.08 ± 177.40	1.90 ± 0.42	0.66 ± 0.25	268.86 ± 90.17	1.93 ± 1.01
WCM + IPE	Caffeic acid	1130.16 ± 162.90	3.32 ± 0.35	0.62 ± 0.14	341.80 ± 123.30	2.82 ± 1.07
	Ferulic acid	34.92 ± 22.61	1.05 ± 0.35	0.33 ± 0.12	26.15 ± 11.58	1.02 ± 0.46
	Protocatechuic acid	193.59 ± 99.99	1.59 ± 0.38	0.41 ± 0.12	121.05 ± 77.40	1.46 ± 0.32
WHM + IPE	Caffeic acid	2301.61 ± 409.95	4.53 ± 1.34	0.93 ± 0.12	709.49 ± 194.27	3.82 ± 2.55
	Ferulic acid	103.90 ± 41.20	2.27 ± 0.42	0.7 ± 0.27	38.75 ± 16.20	1.31 ± 0.32
	Protocatechuic acid	306.73 ± 98.26	1.83 ± 0.24	0.62 ± 0.14	145.46 ± 53.24	1.25 ± 0.36

AUC_0~t_: The area under the drug–time curve; MRT_0~t_: mean residence time; Tmax: the time to peak drug concentration; Cmax: peak concentration; t_1/2_: half-life.

**Table 6:** Effect of IPE on potential biomarkers in serum of WHM and WCM rats.

Group	No.	Metabolite	Formula	HMDB ID	WHM vs. Con				WHM + IPE vs. WHM			
					VIP	Difference Multiple	*p* Value	Trend	VIP	Difference Multiple	*p* Value	Trend
WHM + IPE	1	Urocanic acid	C_6_H_6_N_2_O_2_	0000301	1.008	0.5471	0.001	↓	14.11	309.5	0.000	↑
	2	D-allose	C_6_H_12_O_6_	0001151	7.123	0.6662	0.024	↓	2.675	1.436	0.021	↑
	3	D-mannitol	C_6_H_14_O_6_	0000765	1.555	0.6053	0.000	↓	2.681	5.095	0.000	↑
	4	Hippuric acid	C_9_H_9_NO_3_	0000714	5.063	0.4753	0.000	↓	10.65	10.78	0.000	↑
	5	L-threonate	C_4_H_8_O_5_	0000943	1.954	0.7518	0.001	↓	3.403	3.074	0.000	↑
	6	Pentachlorophenol	C₆HCl₅O	0041974	1.228	0.6585	0.004	↓	1.730	3.064	0.000	↑
	7	Pyrocatechol	C₆H₆O₂	0000957	1.071	0.4134	0.013	↓	2.782	18.23	0.000	↑
	8	1-Methyl-l-histidine	C₇H_11_N₃O₂	0000001	1.469	1.294	0.041	↑	3.027	0.060	0.000	↓
	9	Creatinine	C_4_H_7_N_3_O	0000562	8.835	0.8639	0.012	↓	10.49	1.213	0.000	↑
	10	DL-normetanephrine	C₈H_11_NO₃	0000819	2.890	0.6666	0.036	↓	6.720	3.023	0.000	↑
	11	Malonic acid	C_3_H_4_O_4_	0000691	20.16	0.6487	0.004	↓	22.96	1.609	0.000	↑
WCM + IPE	12	Urocanic acid	C_6_H_6_N_2_O_2_	0000301	1.043	0.7180	0.037	↓	15.12	268.1	0.000	↑
	13	Capric acid	C_10_H_20_O_2_	0000511	1.791	1.312	0.010	↑	2.166	0.4037	0.000	↓
	14	O-succinyl-L-homoserine	C_8_H_13_NO_6_	0255868	1.068	1.262	0.040	↑	1.001	0.6845	0.016	↓
	15	Octanoic acid	C_8_H_16_O_2_	0000482	2.684	1.501	0.008	↑	2.251	0.4895	0.000	↓
	16	p-Coumaryl alcohol	C_9_H_10_O_2_	0003654	1.418	0.6516	0.041	↓	2.614	5.756	0.000	↑
	17	Uracil	C₄H₄N₂O₂	0000300	10.96	1.440	0.000	↑	5.442	0.7988	0.035	↓
	18	γ-Aminobutyric acid	C_4_H_9_NO_2_	0000112	2.308	1.230	0.015	↑	2.497	0.7888	0.008	↓
	19	2′-Deoxycytidine	C_9_H_13_N_3_O_4_	0000014	3.011	1.124	0.011	↑	3.165	0.8917	0.008	↓
	20	Cytosine	C_4_H_5_N_3_O	0000630	4.876	1.114	0.016	↑	4.607	0.9116	0.049	↓
	21	D-mannose	C_6_H_12_O_6_	0000169	2.547	0.5833	0.000	↓	1.072	1.337	0.025	↑

HMDB ID: human metabolome database ID; VIP: variable importance in projection; ↑: up-regulated; ↓: down-regulated.

**Table 7:** Core targets and their degree scores.

Gene	Name	Degree
ALB	Albumin	44
AKT1	AKT serine/threonine kinase 1 Gene	41
EGFR	Epidermal growth factor receptor	37
CASP3	Caspase-3	36

**Table 8:** Mass spectrometry optimization parameters.

Identification	Ion Pair (*m/z*)	Collecting Model	Voltage (V)	Collision Energy (V)
Caffeic acid	179.0350→135.0428	[M−H]^−^	−80	−35
Ferulic acid	193.0506→134.0411	[M−H]^−^	−80	−35
Protocatechuic acid	153.0193→109.0294	[M−H]^−^	−80	−35
Syringic acid	197.0556→123.0091	[M−H]^−^	−80	−20

## Data Availability

The data presented in this study are available on request from the corresponding authors.

## Abbreviations

The following abbreviations are used in this manuscript:

RA	Rheumatoid arthritis
NSAIDS	Nonsteroidal anti-inflammatory drugs
CORT	Corticosteroids
DMARDs	Disease-modifying anti-rheumatic drugs
TNF-α	Tumor Necrosis Factor-α
IL-6	Interleukin- 6
NF-κB	Nuclear Factor kappa-light-chain-enhancer of activated B cells
FLS	Fibroblast-like synoviocytes
CRP	C-reactive protein
RF	Rheumatoid factor
TNF-α	Tumor necrosis factor-α
TGF-β	Transforming growth factor-β
JAK/STAT	Janus kinase/signal transducer and activator of transcription
Akt/mTOR	Akt/mammalian target of rapamycin
LTB4	Leukotriene B4
PI3K/Akt	Phosphatidylinositol 3-kinases/Akt
MAPK	Mitogen-activated protein kinase
JNK	C-Jun N-terminal kinase
ERK	Extracellular regulated protein kinases
C/ebp	CCAAT/enhancer-binding protein
